# Effect of light and prey availability on gene expression of the mixotrophic chrysophyte, *Ochromonas* sp.

**DOI:** 10.1186/s12864-017-3549-1

**Published:** 2017-02-14

**Authors:** Alle A. Y. Lie, Zhenfeng Liu, Ramon Terrado, Avery O. Tatters, Karla B. Heidelberg, David A. Caron

**Affiliations:** Department of Biological Sciences, University of Southern California, 3616 Trousdale Parkway, Los Angeles, CA 90089-0371 USA

**Keywords:** Mixotrophic protist, *Ochromonas*, Gene expression, Phagotrophy, Photosynthesis, Transcriptome

## Abstract

**Background:**

*Ochromonas* is a genus of mixotrophic chrysophytes that is found ubiquitously in many aquatic environments. Species in this genus can be important consumers of bacteria but vary in their ability to perform photosynthesis. We studied the effect of light and bacteria on growth and gene expression of a predominantly phagotrophic *Ochromonas* species. Axenic cultures of *Ochromonas* sp. were fed with heat-killed bacteria (HKB) and grown in constant light or darkness. RNA was extracted from cultures in the light or in the dark with HKB present (Light + HKB; Dark + HKB), and in the light after HKB were depleted (Light + depleted HKB).

**Results:**

There were no significant differences in the growth or bacterial ingestion rates between algae grown in light or dark conditions. The availability of light led to a differential expression of only 8% of genes in the transcriptome. A number of genes associated with photosynthesis, phagotrophy, and tetrapyrrole synthesis was upregulated in the Light + HKB treatment compared to Dark + HKB. Conversely, the comparison between the Light + HKB and Light + depleted HKB treatments revealed that the presence of HKB led to differential expression of 59% of genes, including the majority of genes involved in major carbon and nitrogen metabolic pathways. Genes coding for unidirectional enzymes for the utilization of glucose were upregulated in the presence of HKB, implying increased glycolytic activities during phagotrophy. Algae without HKB upregulated their expression of genes coding for ammonium transporters, implying uptake of inorganic nitrogen from the culture medium when prey were unavailable.

**Conclusions:**

Transcriptomic results agreed with previous observations that light had minimal effect on the population growth of *Ochromonas* sp. However, light led to the upregulation of a number of phototrophy- and phagotrophy-related genes, while the availability of bacterial prey led to prominent changes in major carbon and nitrogen metabolic pathways. Our study demonstrated the potential of transcriptomic approaches to improve our understanding of the trophic physiologies of complex mixotrophs, and revealed responses in *Ochromonas* sp. not apparent from traditional culture studies.

**Electronic supplementary material:**

The online version of this article (doi:10.1186/s12864-017-3549-1) contains supplementary material, which is available to authorized users.

## Background

Mixotrophic protists (phagotrophic phytoflagellates) are a diverse group of protists that can obtain carbon and energy via both phototrophy and heterotrophy. Mixotrophic species possess chloroplasts, but encompass a spectrum of mixotrophic behaviors ranging from nearly purely phototrophic to predominantly phagotrophic due to their ability to consume prey [1]. The extent of these contrasting trophic processes within a species is dependent on both the genetic composition of the mixotroph as well as resource availability in the environment (e.g. light and prey abundance) [2].

The nutritional flexibility of mixotrophic protists complicates efforts to define and model their functional roles in natural communities as they can be both producers and consumers [1–3]. Mixotrophs performing phototrophy contribute to primary production and may compete with other phototrophs for nutrients. On the other hand, phagotrophic activities may provide major nutrients (nitrogen and phosphorus) in excess of cellular needs, which are then released and available for phytoplankton uptake [4, 5]. The activities of mixotrophs and their relationship to other organisms within microbial communities is therefore complex, yet has significant consequences for aquatic food webs and biogeochemical cycles [1, 6, 7]. The important ecological role(s) played by these nutritionally flexible organisms has only been recognized within the last few decades [8, 9].

Understanding the functional role of mixotrophic protists requires knowledge of what trophic activities these organisms are performing within a community, and how they are affected by resource availability. Traditional culture-based studies in the past have documented the trophic tendencies of various mixotrophs [10–13], but have not provided details on the molecular processes occurring in the organisms. More recently, advances in RNA-seq technology have led to studies examining gene expressions of several mixotrophic protists under various environmental conditions that provided inferences about the metabolic processes occurring in response to those conditions [14, 15], although investigations on the effects of specific resources are still rare [16–18]. Such studies may lead to the identification of genes closely associated with a specific trophic mode, which can aid in the challenging goal of determining how mixotrophs are obtaining their nutrition in nature. Hence, this study was performed to examine and compare gene expression by a mixotrophic alga grown under conditions that might induce different trophic activities.

*Ochromonas* is a genus of mixotrophic chrysophyte found in a wide range of aquatic environments, including marine, brackish, freshwater, and even extreme environments such as hypersaline ponds [19, 20] and acidic lakes [21, 22]. Species of *Ochromonas* have been shown to have high bacterial grazing rates, and can be important consumers of bacteria in planktonic communities [5, 22–24], but also employ photosynthesis for survival when prey are insufficient [25, 26]. In addition, species of *Ochromonas* can perform osmotrophy [26], albeit only at high concentrations of labile organic compounds, and have even been shown to grow using phenol as the sole carbon source [27]. The ubiquity, ecological importance, and nutritional flexibility of *Ochromonas* species make them ideal candidates for studying protistan mixotrophy.

We compared the growth and gene expression of a freshwater *Ochromonas* species (strain BG-1) grown under conditions that induced specific trophic modes: phagotrophy (continuous dark with bacteria available), mixotrophy (continuous light with bacteria available), and phototrophy (continuous light after bacteria have been depleted). Pairwise comparisons were made between algae performing phagotrophy vs. mixotrophy and algae performing phototrophy vs. mixotrophy to identify genes that were differentially expressed due to the availability of light or prey, respectively. We also investigated the expression of genes associated with carbon and nitrogen metabolism, as well as tetrapyrrole synthesis. Our study provided molecular details and a better understanding of the metabolic processes of *Ochromonas* sp. under different nutritional modes, thereby demonstrating the usefulness of transcriptomics in understanding the physiology of mixotrophic organisms.

## Methods

### Obtaining and maintaining *Ochromonas* sp.

*Ochromonas* sp. strain BG-1 was kindly provided by Dr. Robert W. Sanders (Temple University, Philadelphia, PA) and was originally isolated from a freshwater pond in Malaysia following organic enrichment in the dark [26]. The alga was subsequently made axenic using antibiotics: 100 mg of penicillin (Sigma-Aldrich, St. Louis, USA) and 50 mg of streptomycin (Sigma-Aldrich) dissolved in 10 ml of ultrapure water (Barnstead GenPure xCAD Plus, Thermo Fisher Scientific, Waltham, USA), and then combined with 20 mg of chloramphenicol (Sigma-Aldrich) dissolved in 0.5 ml of 95% ethanol [26].

Axenic cultures of *Ochromonas* sp. were maintained in a modified DY-V medium [26] (Additional file 1: Table S1) with addition of sterile yeast extract (0.02% final concentration) to support osmotrophic nutrition. The bacterial strain used as prey in the experiment was obtained by streaking bacterized *Ochromonas* sp. culture onto a 1.5% agar plate with 0.5% yeast extract and 0.5% tryptone, and a single colony was subsequently isolated. The isolate was grown for ~4 days in 0.5% yeast extract and 0.5% tryptone broth, heat-killed at 70 °C for 30 min, followed by 3 rounds of centrifugation (10,000 g for 15 min; Sorvall RC5C plus, Thermo Fisher Scientific) and rinsing by resuspension in sterile ultrapure water.

Taxonomic information on the prey bacteria was obtained through its 16S rRNA gene. DNA was extracted from a sample of the bacteria clonal culture using AllPrep DNA/RNA Micro kit (Qiagen, Hilden, Germany). The universal primers 27 F (5’-AGAGTTTGATCMTGGCTCAG-3’) and 1492R (5’-CGGTTACCTTGTTACGACTT-3’) were used for PCR reactions following the procedures detailed in Amutha & Kokila [28]. The amplicons were sent to Genewiz Los Angeles Metro for Sanger sequencing. The 16S rRNA gene sequence was searched against GenBank and found to be a member of the genus *Pseudomonas*. The sequence was deposited in GenBank under the accession number: KY172830.

### Experiment for obtaining *Ochromonas* sp. RNA under different trophic conditions

**Fig. 1:**
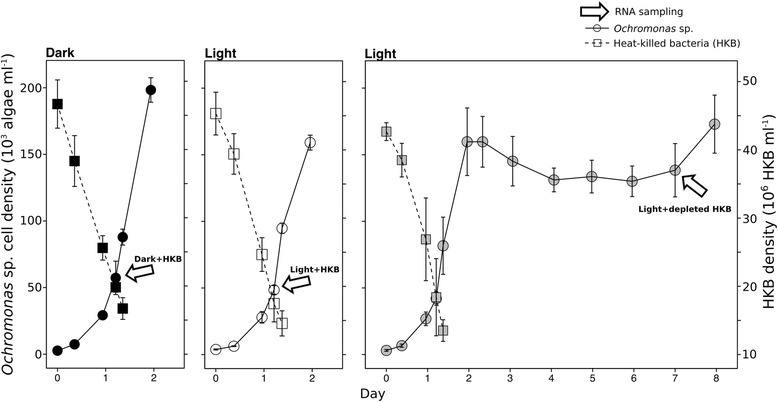
Average abundances (± SD) of *Ochromonas* sp. and heat-killed bacteria (HKB) in different experimental treatments. RNA samples for the Dark + HKB (leftmost panel) and Light + HKB (middle panel) treatments were harvested on day 1, while those for the Light + depleted HKB treatment (rightmost panel) were harvested on day 7

Batch cultures of *Ochromonas* sp. were grown in Fernbach flasks using the modified DY-V medium (Additional file 1: Table S1) without the addition of yeast extract. Cultures were mixed at 70 rpm using magnetic stir bars and incubated at 20 °C under either continuous light at 250 μEinsteins m^-2^ s^-1^ (QSL-100 sensor with QSP-170 deckbox, Biospherical Instruments Inc., San Diego, USA) or in continuous dark (wrapped with aluminum foil). The initial volume of the cultures was 2.5 L, and a one-time dose of heat-killed bacteria (HKB) was added to attain ~ 4.5 x 10^7^ HKB ml^-1^ in all vessels. *Ochromonas* sp. fed with HKB and preacclimated to either the continuous light or dark conditions were used to inoculate experimental cultures at a starting abundance of ~ 4 x 10^3^ algae ml^-1^. Three (triplicate) cultures were incubated in continuous darkness and harvested for RNA during the exponential growth phase (Dark + HKB; day 1 on Fig. 1). Six cultures were incubated in continuous light, with 3 (triplicate) harvested for RNA during the exponential growth phase (the same sampling time as the cultures in continuous darkness; Light + HKB; day 1 on Fig. 1), and 3 (triplicate) harvested approximately 5 days after the depletion of HKB (Light + depleted HKB; day 7 on Fig. 1). While this species of *Ochromonas* can survive using only phototrophy after the depletion of HKB, it does not survive in constant darkness without HKB (Detailed in discussion). A dark treatment without HKB was thus not included in the experiment as it would consist of starving cells without any means of carbon or energy acquisition. Axenicity tests were performed at the beginning and end of the experiment (day 8) for all treatments and replicates by adding 5 ml aliquots to 7 ml of 0.5% yeast extract and 0.5% tryptone broth. Cultures were deemed axenic if no growth of bacteria or fungi was observed after two weeks.

### Samples for cell abundances and chlorophyll

Samples were taken periodically to determine the growth rates of *Ochromonas* sp. and their rates of ingestion on HKB. *Ochromonas* sp. abundance was estimated from 1 ml samples preserved with acidic Lugol’s solution (final concentration 5%) that were enumerated using a Palmer-Maloney counting chamber and a compound microscope (200 x; counted to ~300 cells or a maximum of 100 fields of view; BX51; Olympus, Waltham, USA). Cultures harvested for RNA during the exponential growth phase on day 1 (i.e. Light + HKB and Dark + HKB treatments) were maintained in experimental conditions for the duration of the experiment (8 days), and sampled for *Ochromonas* sp. abundance until day 2 for growth rate estimation. The exponential growth rate of the alga was calculated as the slope of natural log algal abundance against time using the linear portion of the curve (day 0 – 2; Additional file 2: Figure S1). Aliquots (0.1 – 1 ml) of samples for HKB abundance (4.5 ml culture preserved with formaldehyde at 1% final concentration) were stained with 4' 6-diamidino-2-phenylindole dihydrochloride (DAPI; 300 μl of 0.1 μg/μl; Sigma-Aldrich) and filtered onto a 0.2 μm black polycarbonate filters (25 mm, Whatman, Maidstone, UK). Counts for HKB (counted to ~300 cells or a maximum of 100 fields of view) were performed at 100 x on a compound microscope equipped with epifluorescence illumination (BX51; Olympus). Grazing rates of HKB were calculated from the first and last time points for HKB enumeration (day 0 – 1; Fig. 1) using the formula:

$$ \left({\left[\mathrm{HKB}\right]}_{\mathrm{final}}\hbox{--}\ {\left[\mathrm{HKB}\right]}_{\mathrm{initial}}\right)/\left(\left[\mathrm{Average}\ \mathrm{Algae}\right]\ \mathrm{x}\ \mathrm{Change}\ \mathrm{in}\ \mathrm{time}\right) $$

in which average algal abundance is calculated as:

$$ \left({\left[\mathrm{Algae}\right]}_{\mathrm{final}}\hbox{--}\ {\left[\mathrm{Algae}\right]}_{\mathrm{initial}}\right)/\left(\mathrm{LN}\left({\left[\mathrm{Algae}\right]}_{\mathrm{final}}/{\left[\mathrm{Algae}\right]}_{\mathrm{initial}}\right)\right). $$

Additional samples for HKB were collected daily after day 2 to check for the presence of bacterial cells.

Samples for chlorophyll *a* concentration measurements were collected when algae were harvested for RNA extraction. Aliquots (50 – 75 ml) of cultures were filtered onto GF/F filters (25 mm, Whatman) and stored at -80 °C until extraction. Chlorophyll *a* was extracted using 100% acetone (Sigma-Aldrich) for 24 h in the dark at -20 °C, and fluorescence was measured on a fluorometer (Trilogy; Turner Designs, San Jose, USA) using a non-acidification method [29].

### Extraction and sequencing of RNA

Algae harvested for RNA (800 ml aliquots) were concentrated by centrifugation (5,000 g for 10 min; Sorvall RC5C plus). Pellets were preserved in 0.75 ml of RNAlater (Sigma-Aldrich) immediately after centrifugation and stored at -20 °C until RNA extraction. RNA was extracted using RNeasy Plant Mini Kit (Qiagen) followed by DNA removal using DNase (Sigma-Aldrich). The absence of genomic DNA contamination was verified by PCR reactions on the 18S rRNA gene using V4 primers (Forward: 5’-CCAGCA[GC]C[CT]GCGGTA ATTCC-3; Reverse: 5’- ACTTTCGTTCTTGAT[CT][AG]A-3’) following procedures described in Hu et al. [30]. Total RNA was cleaned and concentrated with RNA Clean & Concentrator kit (Zymo Research, Irvine, USA). cDNA library construction and sequencing was carried out at the University of Southern California Epigenome Center on Illumina Hi-Seq 2000 (100 bp paired-end). RNA was first cleaned with Agencourt AMPure XP magnetic beads (volume ratio of 0.8 x; Beckman Coulter, Indianapolis, USA) to remove low molecular weight material (mostly denatured HKB RNA), and then 500 ng of RNA was used for library construction. RNA samples were spiked with ERCC standards as per manufacturer's instructions (Ambion LifeTech/Thermo Fisher Scientific, Waltham, USA), and TruSeq v2 mRNA kit (Illumina, San Diego, USA) was used for library construction. ERCC standards were added with the intention of estimating the absolute abundances of mRNA per alga [31], but the presence of large amounts of HKB in the Light + HKB and Dark + HKB treatments led to unanticipated decreases in RNA extraction efficiencies. As a result, estimates of RNA yield per algae were unreliable, and results from the ERCC standards were not used in our analyses. Raw sequences generated were deposited in the NCBI Sequence Read Archive under the accession numbers: SRX965527 (Light + HKB), SRX968591 (Light + depleted HKB), and SRX970036 (Dark + HKB).

### Transcriptome processing

The transcriptome was assembled *de novo* with all 9 libraries (3 treatments x 3 replicates) using Trinity [32]. Script align_and_estimate_abundance.pl included in Trinity (with RSEM [33] and bowtie [34]) was used to estimate fragment per kilobase of transcript per million reads (FPKM) values and percentages of isoform support. Transcripts with < 1% isoform support and < 1 FPKM in all libraries were discarded. TransDecoder [35] was used to identify potential coding regions within reconstructed transcripts. CD-HIT-EST [36] was used to remove identical redundant coding genes. Putative genes were annotated using protein databases, including BLAST searches against NCBI nr database, and HMMER [37] against Tigrfam and Pfam databases (e-value cutoff: < 10^-5^). Genes associated with different metabolic processes were identified using KAAS [38], and additional functional annotation obtained from Blast2GO (version 4.0.7; e-value cutoff: < 10^-5^) [39] is provided in the supplementary materials. All annotations of genes specifically mentioned in results and discussion were manually curated based on the combination of all database searches. Absence of certain genes were manually confirmed by BLAST searches of such genes from other organisms against the entire transcriptome. Genes that did not have > 1 FPKM in at least 2 libraries were not considered in this study. If the FPKM of a gene was 0 in a library as a result of 0 fragment count, then the fragment count was replaced with 1 to allow downstream processing e.g. the calculation of fold changes.

The statistical software package edgeR (version 3.10.5) was used to identify genes that were differentially expressed between different treatments [40]. All mentions of ‘upregulation’ or ‘differential expression’ in the following sections thus refer to genes with statistically significant differential expression (false-discovery rate < 0.05). There were 2 major pairwise comparisons in this study: i) Dark + HKB vs. Light + HKB; and ii) Light + HKB vs. Light + depleted HKB. Differences in gene expression in the first comparison were due to the availability of light, while differences in the latter comparison were due to the availability of HKB and potentially to differences in life stage. Results from edgeR were compared and validated with results from DeSeq2 (version 3.4), an independent statistical software for gene differential expression analysis [41].

Chloroplast and mitochondrial genes in the transcriptome were excluded from differential expression analysis as they may be non-polyadenylated RNA that bypassed polyadenylation selection (included in the mRNA kit) or polyadenylated organelle genes marked for degradation [42, 43]. Chloroplast genes in the transcriptome were identified by aligning genes to chloroplast genomes of 14 stramenopiles (Additional file 3: Table S2). An *Ochromonas* sp. gene was marked as a chloroplast gene if it aligned to genes in > 6 of the chloroplast genomes (e-value cutoff: 10^-5^, > 50% amino acid identity). Mitochondrial genes in the transcriptome were identified by alignment to the mitochondrial genome of *Ochromonas danica* (e-value cutoff: < 10^-5^, > 50% amino acid identity; NCBI accession number: NC_002571).

## Results

### Growth experiment

The exponential growth rates of *Ochromonas* sp. (calculated from day 0 – 2) were similar for cultures grown in light (2.1 ± 0.1 d^-1^) and in dark (2.3 ± 0.1 d^-1^; One-way ANOVA *p* > 0.05; Fig. 1). Light did not support population growth of *Ochromonas* sp. once HKB were depleted (after day 2). Grazing rates of the alga on HKB (calculated from day 0 – 1) were also similar for cultures grown in light (38 ± 8 HKB h^-1^ grazer^-1^) and in dark (37 ± 3 HKB h^-1^ grazer^-1^; One-way ANOVA *p* > 0.05; Fig. 1). Low HKB abundances (< 10^6^ HKB ml^-1^) and high *Ochromonas* sp. abundances (> 10^5^ HKB ml^-1^) after day 1 made the enumeration of HKB difficult due to the accumulation of cellular debris. HKB abundances were therefore not quantified after day 1, but daily samples were inspected for the presence of bacteria through day 8. No bacterial cells were observed in 1 ml of any sample after day 2. Axenicity tests performed on the last day of the experiment indicated that one of the replicates in the Light + HKB treatment was contaminated with live bacteria on day 8, but it is uncertain when the contamination occurred.

Chlorophyll *a* content per alga at the time of RNA sampling on day 1 (Fig. 1) in the Light + HKB treatment (5.5 ± 1.6 fg alga^-1^) was not significantly different than that of the Dark + HKB treatment (1.6 ± 0.4 fg alga^-1^; One-way ANOVA, *p* > 0.05). The chlorophyll *a* content per alga in the Light + depleted HKB treatment at the time of RNA sampling on day 7 (14.1 ± 3.8 fg alga^-1^) was significantly higher compared to values of both the Light + HKB and Dark + HKB treatments that were sampled on day 1 (One-way ANOVA, *p* < 0.05).

### Transcriptome overview

The *de novo* transcriptome of *Ochromonas* sp. assembled from all 9 libraries yielded a transcriptome size of 28 Mbp. It consisted of 21,472 genes, but the removal of organelle genes and genes that did not have > 1 FPKM in at least 2 libraries reduced the total number of genes to 18,154. More than 25% (4,878) of the remaining genes did not have any matches in public databases, and an additional 17% (3,155 genes) matched to hypothetical or unknown proteins. The Light + HKB treatment replicate (Light + HKB A in Additional file 4: Figure S2) that demonstrated contamination with live bacteria on day 8 clustered closely with an uncontaminated replicate of the same treatment on a non-metric multidimensional scaling analysis of the transcriptome libraries (Additional file 4: Figure S2), and was thus kept in all analyses.

A small portion of genes (8% of all genes in transcriptome) was found to be differentially expressed between the Dark + HKB and Light + HKB treatments using edgeR, with almost twice as many upregulated in Light + HKB than those upregulated in Dark + HKB (Table 1). On the other hand, more than half of the genes in the transcriptome (59% of all genes) were differentially expressed between the Light + HKB and Light + depleted HKB treatments, with similar numbers upregulated in each treatment (Table 1). DeSeq2 produced similar results in which light and HKB led to 8% and 62% of genes differentially expressed respectively. Only results from edgeR are reported for the following sections.

**Table 1:** Number of differentially expressed genes in the pairwise comparisons of Dark+HKB vs. Light+HKB (left 2 columns) and Light+HKB vs. Light+depleted HKB (right 2 columns)

	Dark+HKB vs. Light+HKB		Light+HKB vs. Light+depleted HKB	
	Upregulated in Dark+HKB	Upregulated in Light+HKB	Upregulated in Light+HKB	Upregulated in Light+depleted HKB
Total number of differentially expressed genes	471	906	5,540	5,152
Number of differentially expressed phototrophy-related genes	1	16	7	7
Number of differentially expressed phagotrophy-related genes	0	20	71	24
Number of differentially expressed genes coding for glycoside hydrolases and lysozymes	0	16	32	14
Number of differentially expressed genes involved in major carbon metabolic pathways	3	8	59	18
Number of differentially expressed genes involved in major nitrogen metabolic pathways	5	0	7	12
Number of differentially expressed genes involved in tetrapyrrole synthesis	0	15	18	12

### Differential expression of phototrophy-related genes

We defined phototrophy-related genes as genes involved in 3 KEGG ortholog groups: Photosynthesis (KO00195); Photosynthesis antenna proteins (KO00196); and Carotenoid biosynthesis (KO00906). The group ‘Photosynthesis’ includes genes associated with photosystems, while genes in the other two groups are involved in light harvesting and pigment production. The KEGG ortholog groups ‘Carbon fixation’ (KO00710) and ‘Porphyrin and chlorophyll metabolism’ (KO00860) are also related to phototrophy, but genes in these groups are not included in this section as they are detailed in following sections (Carbon metabolism; and Tetrapyrrole synthesis).

**Fig. 2:**
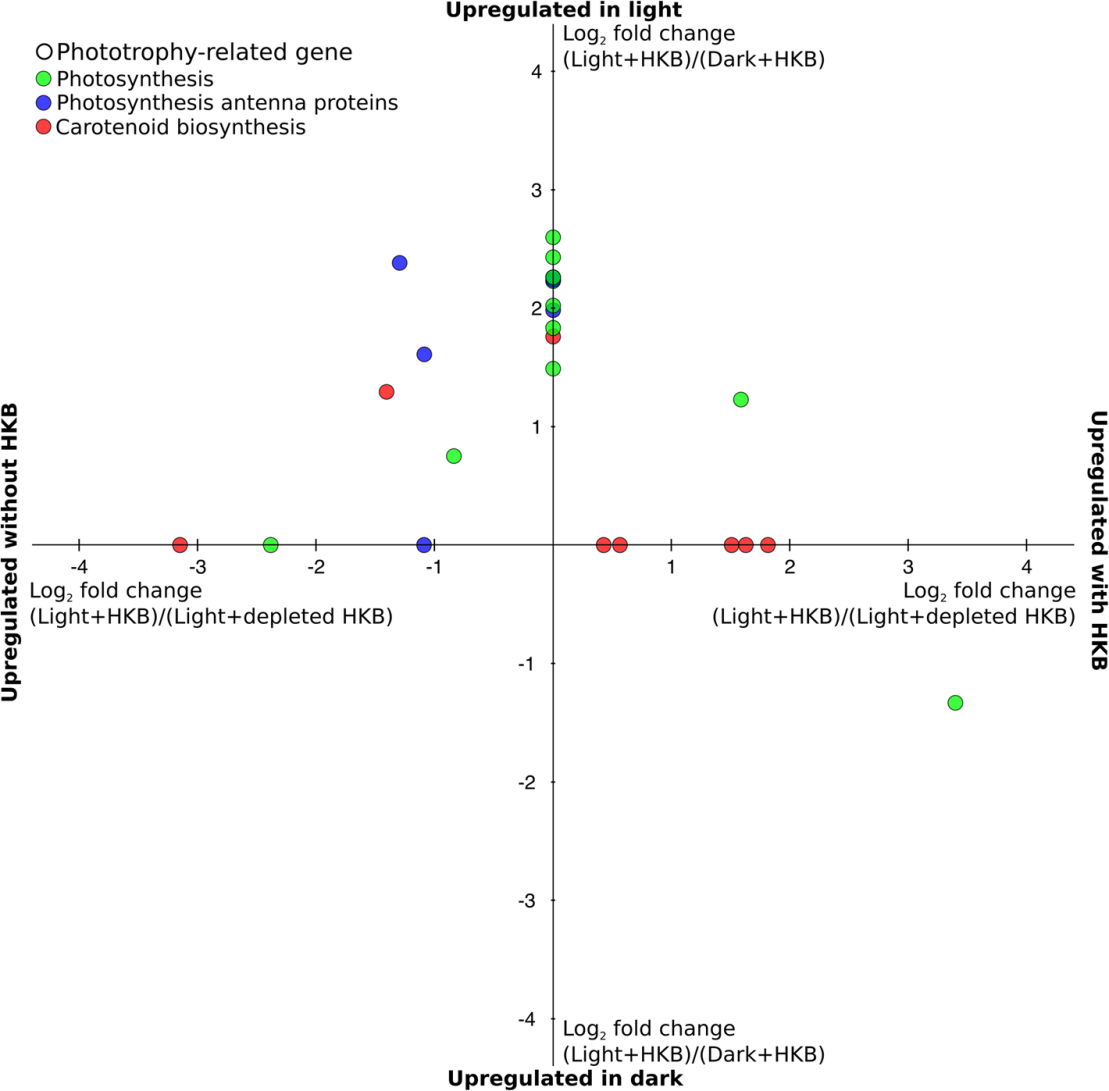
Average log_2_ fold change of differentially expressed phototrophy-related genes of *Ochromonas* sp. in the pairwise comparisons: Light + HKB vs. Dark + HKB (y-axis); and Light + HKB vs. Light + depleted HKB (x-axis). Values above the x-axis indicate genes upregulated in the Light + HKB treatment compared to the Dark + HKB treatment, while values to the right of the y-axis indicate genes upregulated in the Light + HKB treatment compared to the Light + depleted HKB treatment. Values on the x-axis indicate genes not differentially expressed between the Dark + HKB and Light + HKB treatments, while values on the y-axis indicate genes not differentially expressed between the Light + HKB and Light + depleted HKB treatments. Colors indicate the KEGG ortholog group of the gene

A total of 26 phototrophy-related genes were identified in the transcriptome, 16 of which were upregulated in the Light + HKB treatment compared to Dark + HKB, including the majority of genes associated with photosystems or antenna proteins (data points above the x-axis of Fig. 2; Additional file 5: Table S3). There was a ferredoxin associated with photosynthesis that was upregulated in Dark + HKB compared to Light + HKB (data point below the x-axis of Fig. 2; Additional file 5: Table S3).

Comparison of the Light + HKB and Light + depleted HKB treatments revealed upregulation of 7 phototrophy-related genes in Light + HKB (data points to the right of the y-axis of Fig. 2) and also 7 genes upregulated in Light + depleted HKB (data points to the left of the y-axis of Fig. 2). Half of the genes coding for antenna proteins were upregulated in the Light + depleted HKB treatment and the remaining were not differentially expressed between the 2 treatments (Fig. 2; Additional file 5: Table S3).

### Differential expression of phagotrophy-related genes

**Fig. 3:**
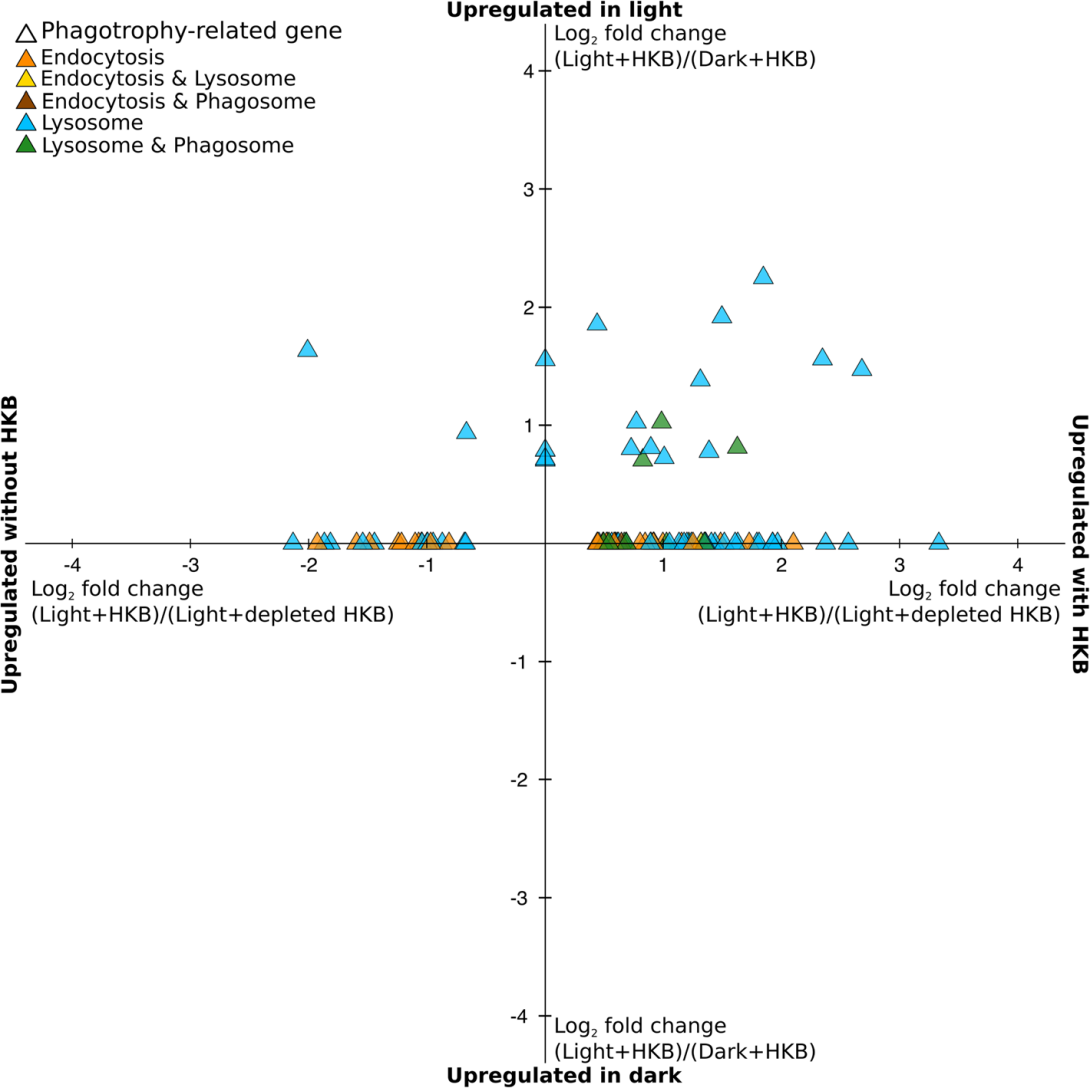
Average log_2_ fold change of differentially expressed phagotrophy-related genes of *Ochromonas* sp. in the pairwise comparisons: Light + HKB vs. Dark + HKB (y-axis); and Light + HKB vs. Light + depleted HKB (x-axis). Values above the x-axis indicate genes upregulated in the Light + HKB treatment compared to the Dark + HKB treatment, while values to the right of the y-axis indicate genes upregulated in the Light + HKB treatment compared to the Light + depleted HKB treatment. Values on the x-axis indicate genes not differentially expressed between the Dark + HKB and Light + HKB treatments, while values on the y-axis indicate genes not differentially expressed between the Light + HKB and Light + depleted HKB treatments. Colors indicate the KEGG ortholog group(s) of the gene

A total of 142 genes in the transcriptome were associated with Endocytosis (KO04144), Phagosomes (KO04145), or Lysosomes (KO04142), as identified by their KEGG orthology annotation (Additional file 5: Table S3). None of these genes potentially related to phagotrophy were upregulated in Dark + HKB when compared to Light + HKB (no data point below the x-axis of Fig. 3). In contrast, 20 phagotrophy-related genes were upregulated in the Light + HKB treatment compared to Dark + HKB (data points above the x-axis of Fig. 3), 18 of which were catabolic enzymes such as proteases (Additional file 5: Table S3).

There was upregulation of 71 phagotrophy-related genes in the Light + HKB treatment compared to the Light + depleted HKB treatment (data points to the right of the y-axis of Fig. 3), and 24 phagotrophy-related genes upregulated in the Light + depleted HKB treatment (data points to the left of the y-axis in Fig. 3). The majority of catabolic enzymes and vacuolar ATPases were upregulated in Light + HKB compared to Light + depleted HKB (Additional file 5: Table S3).

Combining the results of the pairwise comparisons between i) Dark + HKB vs. Light + HKB; and ii) Light + HKB vs. Light + depleted HKB revealed 14 lysosomal genes that had significantly higher expression when both light and HKB were available (upper right quadrant of Fig. 3). These genes included 1 glycoside hydrolase and 13 proteases (Additional file 5: Table S3).

In addition to genes involved in phagosomes, lysosomes, or endocytosis identified by KEGG, we also investigated the expression of genes specifically coding for glycoside hydrolases and lysozymes. There were 68 genes for glycoside hydrolases and 4 genes for lysozymes identified in the transcriptome based on their annotations from GenBank, Tigrfam, and Pfam (Additional file 5: Table S3). Sixteen genes for glycoside hydrolases were upregulated in Light + HKB compared to Dark + HKB (Table 1), and 13 of these 16 glycoside hydrolase genes were also upregulated in Light + HKB compared to Light + depleted HKB, indicating that their expression was highest in the Light + HKB treatment out of all three treatments (Additional file 5: Table S3). Comparisons between the Light + HKB and Light + depleted HKB treatments revealed 31 glycoside hydrolase genes upregulated in Light + HKB and 11 upregulated in Light + depleted HKB (Table 1). Among the 4 lysozyme genes identified in the transcriptome, none were differentially expressed between the Dark + HKB and Light + HKB treatments, while 1 was upregulated in the Light + HKB treatment and 3 were upregulated in Light + depleted HKB when comparing these two treatments (Additional file 5: Table S3).

### Carbon metabolism

**Fig. 4:**
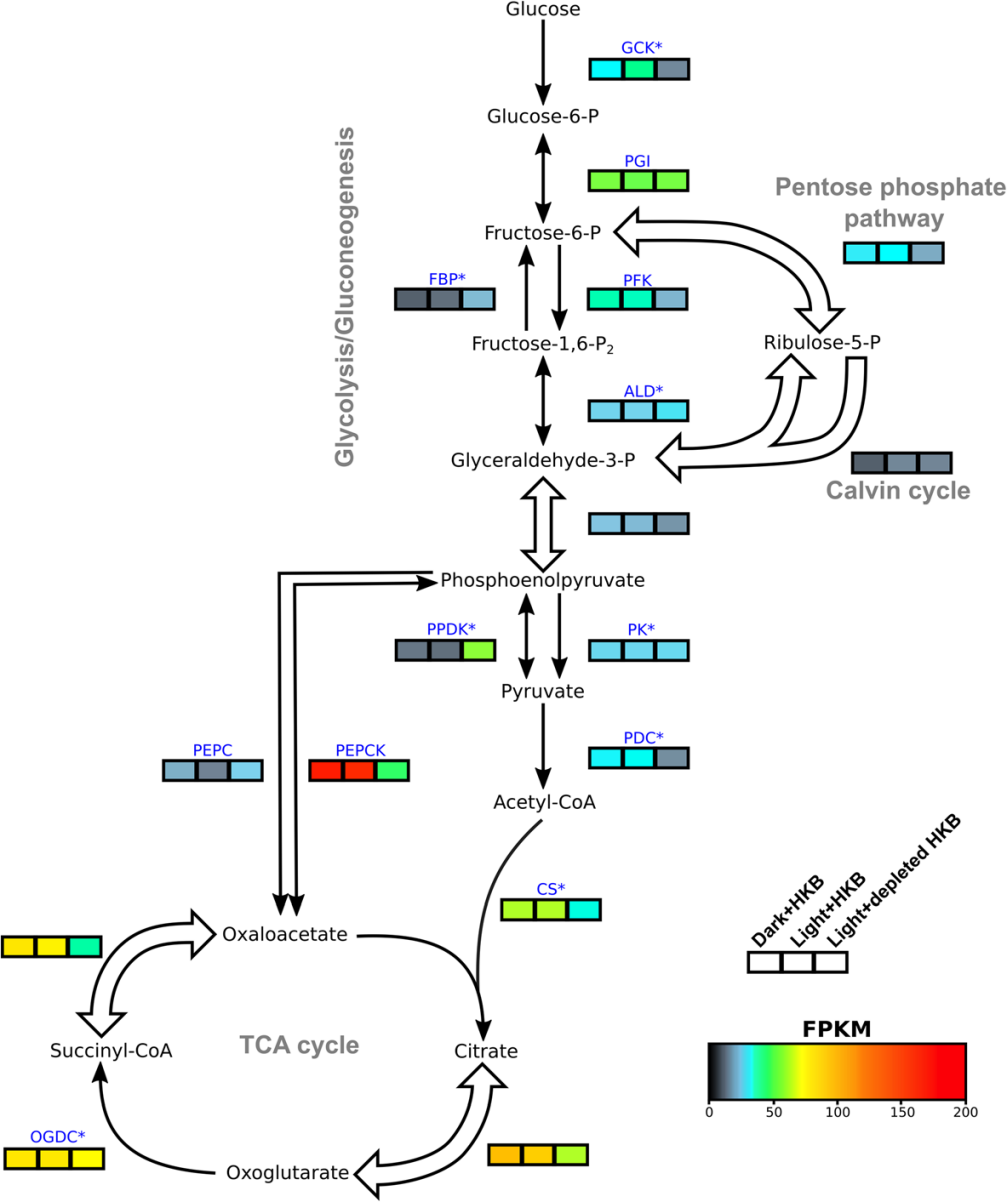
A heatmap of gene expression (fragment per kilobase of transcript per million reads (FPKM)) for enzymes involved in major carbon metabolic pathways in *Ochromonas* sp. in different treatments. Expression for each treatment was the average between the 3 replicates. Blue letters indicate enzyme abbreviations (Refer to Additional file 7: Table S5 for the full name of each enzyme). An asterisk next to the enzyme abbreviation indicates there were multiple paralogs for the enzyme, in which case the expression values were calculated as the geometric means of all paralogs (Refer to Additional file 6: Table S4 for the expression values of each paralog). Block arrows indicate pathways that involve multiple enzymes, and expression values were summarized as the geometric means of all enzymes and their paralogs in the pathways (Refer to Additional file 6: Table S4 for the expression values of each gene and paralog). Only genes for enzymes exclusive to the Calvin cycle (i.e. ribulose-1,5-bisphosphate carboxylase/oxygenase, sedoheptulose bisphosphatase, and phosphoribulokinase) were categorized as “Calvin cycle”

A total of 107 genes in the transcriptome were involved in glycolysis/gluconeogenesis, non-oxidative pentose phosphate pathway, Calvin cycle, and tricarboxylic acid (TCA) cycle (Additional file 6: Table S4), and only 10% of these genes were differentially expressed between the Dark + HKB and Light + HKB treatments (Fig. 4; Table 1). There were 3 genes upregulated in Dark + HKB and 8 genes upregulated in Light + HKB when comparing these two treatments, with 4 of the 8 genes upregulated in Light + HKB involved in the Calvin cycle (Additional file 6: Table S4).

The comparison between the Light + HKB and Light + depleted HKB treatments, on the other hand, revealed differential expression of 72% of genes associated with major carbon metabolic pathways (Fig. 4). There were 59 genes upregulated in Light + HKB, including genes of unidirectional enzymes for the utilization of glucose, such as glucokinase (GCK) and phosphofructokinase (PFK; Fig. 4; Additional file 6: Table S4). Among the 18 genes that were upregulated in Light + depleted HKB, 6 were involved in the pentose phosphate pathway and Calvin cycle (Additional file 6: Table S4). Many enzymes in the Calvin cycle are also involved in the pentose phosphate pathway (e.g. transaldolase and transketolase), so genes for enzymes overlapping both pathways were categorized as ‘pentose phosphate pathway’ in Fig. 4, and only those exclusive to the Calvin cycle (i.e. ribulose-1,5-bisphosphate carboxylase/oxygenase, sedoheptulose bisphosphatase, and phosphoribulokinase) were categorized as ‘Calvin cycle’. The gene for phosphoenolpyruvate carboxylase (PEPC) was upregulated in Light + depleted HKB, while the gene for phosphoenolpyruvate carboxykinase (PEPCK) was upregulated in Light + HKB when comparing these two treatments (Fig. 4).

### Nitrogen metabolism

**Fig. 5:**
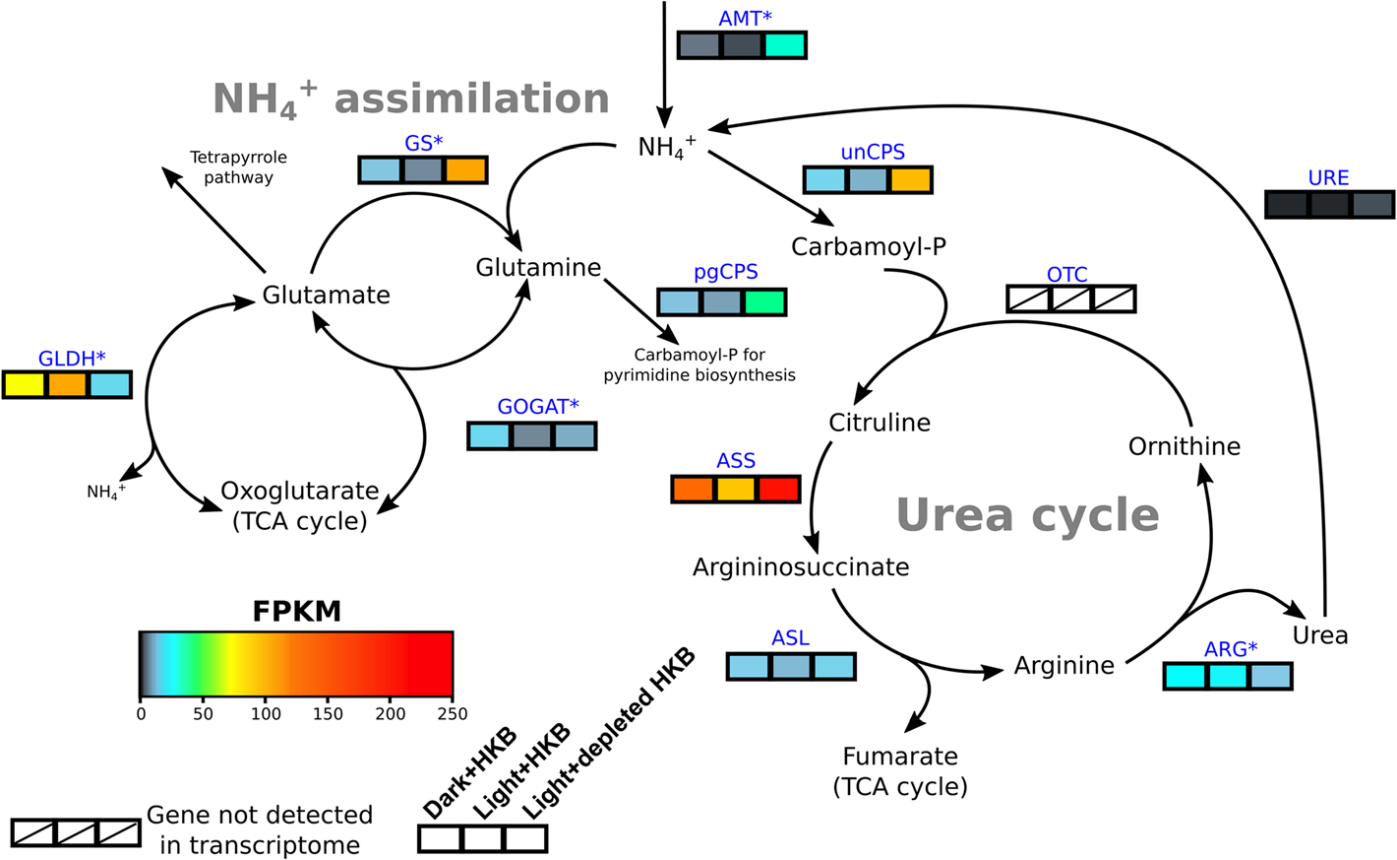
A heatmap of gene expression (fragment per kilobase of transcript per million reads (FPKM)) for enzymes involved in ammonium assimilation and urea cycle in *Ochromonas* sp. in different treatments. Expression for each treatment was the average between the 3 replicates. Blue letters indicate enzyme abbreviations (Refer to Additional file 7: Table S5 for the full name of each enzyme). An asterisk next to the enzyme abbreviation indicates there were multiple paralogs for the enzyme, in which case the expression values were calculated as the geometric means of all paralogs (Refer to Additional file 6: Table S4 for the expression values of each paralog)

A total of 20 genes involved in ammonium assimilation and the urea cycle were identified (Additional file 6: Table S4), while genes associated with urea transport and the uptake and assimilation of nitrate (i.e. nitrate transporter, nitrite transporter, nitrate reductase, and nitrite reductase) were absent from the transcriptome. Genes for ornithine transcarbamylase (OTC) in the urea cycle were also not detected in the transcriptome (Fig. 5). There were 5 genes associated with these major nitrogen metabolic pathways upregulated in Dark + HKB and none upregulated in Light + HKB when comparing these two treatments (Fig. 5; Additional file 6: Table S4). However, the majority of genes associated with ammonium assimilation and urea cycle (95%) were differentially expressed between the Light + HKB and Light + depleted HKB treatments (Fig. 5; Additional file 6: Table S4). Most ammonium transporter (AMT) genes were upregulated in Light + depleted HKB (5 of 7 AMT genes), but the remaining 2 were upregulated in Light + HKB (Additional file 6: Table S4). All glutamine synthetase genes (GS) were upregulated in Light + depleted HKB, while all glutamate dehydrogenase (GLDH) genes were upregulated in Light + HKB (Fig. 5; Additional file 6: Table S4).

### Tetrapyrrole synthesis

**Fig. 6:**
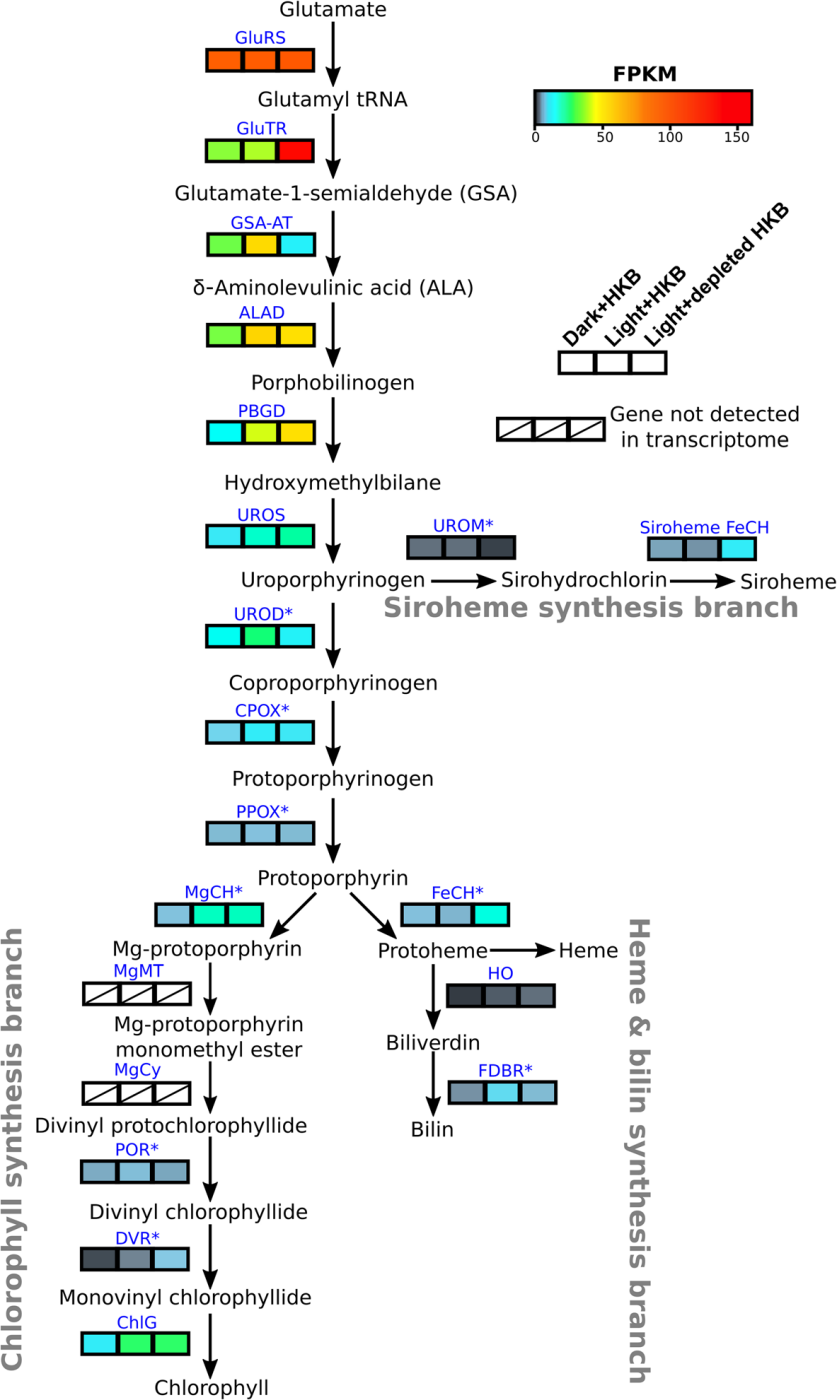
A heatmap of gene expression (fragment per kilobase of transcript per million reads (FPKM)) for enzymes involved in tetrapyrrole synthesis in *Ochromonas* sp. in different treatments. Expression for each treatment was the average between the 3 replicates. Blue letters indicate enzyme abbreviations (Refer to Additional file 7: Table S5 for the full name of each enzyme). An asterisk next to the enzyme abbreviation indicates that there were multiple paralogs for the enzyme, in which case the expression value was calculated as the geometric mean of expression for all paralogs (Refer to Additional file 6: Table S4 for the expression values of each paralog)

There were 50 genes involved in tetrapyrrole synthesis in the transcriptome (Fig. 6; Additional file 6: Table S4). Genes coding for δ-aminolevulinic acid synthase (required for the C4 tetrapyrrole synthesis pathway typically utilized by heterotrophic eukaryotes) were not observed in the transcriptome, but genes were found for glutamyl-tRNA reductase (GluTR) and glutamate 1-semialdehyde aminotransferase (GSA-AT), which are required for the C5 pathway typically present in photosynthetic organisms. Genes for magnesium protoporphyrin IX methyltransferase (MgMT) and magnesium-protoporphyrin-IX-monomethyl ester cyclase (MgCy) that are involved in the chlorophyll synthesis branch were also not detected in the transcriptome (Fig. 6). A number of genes associated with tetrapyrrole synthesis, including δ-aminolevulinic acid dehydratase (ALAD), porphobilinogen deaminase (PBGD), and chlorophyll synthase (ChlG), responded to the presence of light as they were upregulated in cultures grown in the light compared to cultures grown in the dark (i.e. upregulated in Light + HKB or Light + depleted HKB compared to Dark + HKB; Fig. 6; Additional file 6: Table S4).

## Discussion

### The nutritional strategy of *Ochromonas* sp. strain BG-1

Our experiments confirmed previous studies that showed *Ochromonas* sp. strain BG-1 is a predominantly phagotrophic mixotroph [26]. The alga had a grazing rate of ~40 bacteria alga^-1^ h^-1^ that was similar to those reported for the same strain in another study [26], and at the higher end of the range of values reported for other species of *Ochromonas*, *Poterioochromonas* (a closely related mixotrophic genus), and similar-sized phagotrophic flagellates [22, 44–47]. Light did not have a significant effect on the bacterial grazing rate of strain BG-1, unlike other species of *Ochromonas* or *Poterioochromonas* that have been found to have increased grazing rate in the dark [48–50], or other species of mixotrophs that appear to increase their grazing rates with increasing light [51].

Light also did not have a significant effect on the population growth of *Ochromonas* sp. strain BG-1 in the presence of prey, as the exponential growth rates of the alga were indistinguishable between cultures grown with or without light (Fig. 1). Indeed, Terrado et al. [52] demonstrated a low contribution of fixed inorganic carbon (1 - 11%) to the total carbon content of *Ochromonas* sp. strain BG-1 with access to both light and bacterial prey. However, we observed that this species of *Ochromonas* maintained its population abundance after >5 months following the depletion of HKB when light was supplied, while cultures kept in continuous darkness had a significant decrease in abundance several weeks after the depletion of HKB (data not shown). These observations are in agreement with previous conjecture that photosynthesis is primarily a survival mechanism in this predominantly phagotrophic alga [10, 25, 53, 54].

### The expression of genes related to phototrophy and phagotrophy

Light did not augment the growth of *Ochromonas* sp. strain BG-1 in the presence of bacterial prey, yet there was upregulation of a number of genes associated with photosynthesis in the Light + HKB treatment compared to the Dark + HKB treatment (data points above the x-axis of Fig. 2). The upregulation of these genes implied increased synthesis of some, but not all components of the photosynthetic machinery in algae growing with both light and prey. Specifically, chlorophyll *a* content per alga in the Light + HKB treatment was significantly lower than that in the Light + depleted HKB treatment, suggesting that the light harvesting capability of *Ochromonas* sp. strain BG-1 was reduced in the presence of prey. Lower chlorophyll *a* content has been reported in *Poterioochromonas malhamensis* in the presence of bacterial prey compared to conditions when bacteria were depleted [53]. Increased chlorophyll *a* content in the Light + depleted HKB treatment in this study coincided with upregulation of genes associated with light harvesting (e.g. antenna proteins) and chlorophyll synthesis (e.g. GluTR) compared to the Light + HKB treatment (data points to the left of the y-axis of Fig. 2; Fig. 6; Additional file 5: Tables S3 and Additional file 6: Table S4).

We hypothesized that reduction in light harvesting capability and efficiency of the photosynthetic machinery in the presence of prey may occur to minimize photooxidative stress and the production of excess reducing agent (NADPH) in the alga while energy and carbon are readily available via phagotrophy. Intense photosynthetic activities under mixotrophic conditions would presumably lead to the accumulation of excess NADPH, because the reducing agent would be generated from both the light reaction of photosynthesis and phagotrophic reactions. For example, the expression of the gene for glucose-6-phosphate dehydrogenase, which generates NADPH from the breakdown of glucose-6-phostphate, was upregulated in the presence of HKB (upregulated in Light + HKB vs. Light + depleted HKB). Such an accumulation in reducing power can result in the over-excitation of the photosynthetic machinery, necessitating adjustments to the light harvesting units for the balancing of the light-dependent and independent (i.e. Calvin cycle) reactions of photosynthesis [55].

There was upregulation of a number of genes potentially related to phagotrophy in the Light + depleted HKB treatment compared to the Light + HKB treatment (data points to the left of the y-axis of Fig. 3), despite the depletion of prey and thus the lack of grazing activities after day 2. This finding was contrary to our expectations, but the identity and knowledge of phagotrophy-related genes are currently largely based on studies of macrophages [56–58], and it is possible that genes associated with phagocytosis in higher animals may not have the same function in protists [59]. In addition, the depletion of HKB in the Light + depleted HKB treatment led to a significant decrease in growth rate (from 2.1 d^-1^ to 0.0 d^-1^), and algae without bacterial prey may undergo autophagy for the internal recycling of nutrients [60], a process that also involves lysosomes [61]. Hence, it is uncertain that genes identified as phagotrophy-related in this study are truly or solely associated with phagotrophy in *Ochromonas* sp. strain BG-1. It is also possible that high algal abundances during the late stages of the experiment led to cannibalism, which has been documented in various *Ochromonas* species [10, 62, 63]. However, we did not observe a bimodal distribution of cell sizes (data not shown) indicative of cannibalism in protistan cultures in our experiment [64].

The expression of glycoside hydrolases and lysozymes also did not appear to be specifically associated with the occurrence of phagotrophic activities of *Ochromonas* sp. strain BG-1. Glycoside hydrolases are enzymes that hydrolyze the glycosidic bond between a carbohydrate and other molecules, and the digestion of bacteria is among one of their wide range of functions [65]. Lysozyme is a type of glycoside hydrolase with the specific function of digesting bacterial cell walls [66]. Surprisingly, the majority of genes coding for lysozymes and a number of glycoside hydrolase genes were upregulated when prey were depleted (i.e. upregulated in Light + depleted HKB compared to Light + HKB; Additional file 5: Table S3). The reasons for this phenomenon could be among the two previously mentioned for the upregulation of potentially phagotrophy-related genes in the Light + depleted HKB treatment compared to Light + HKB (i.e. genes not specifically associated with phagotrophy; or cannibalism).

Interestingly, the expression of a number of genes for lysosomal proteases and glycoside hydrolases were highest in the Light + HKB treatment (i.e. upregulated in Light + HKB compared to Dark + HKB, as well as in Light + HKB compared to Light + depleted HKB; upper right quadrant of Fig. 3; Additional file 5: Table S3), indicating they were upregulated in response to the presence of both light and bacteria. As lysosomes and glycoside hydrolases are involved in various cellular processes in addition to prey digestion [58, 65], these catabolic enzymes upregulated in the presence of both light and prey may be involved in the metabolism of different metabolites that are unique to mixotrophic nutrition. Boéchat et al. [48], for example, demonstrated that *Ochromonas* sp. with access to both light and prey had a distinct set of fatty acids compared to those with access to only light or prey, and suggested that there were synergistic effects when both light and prey were supplied to *Ochromonas* sp.

### The expression of genes associated with major carbon and nitrogen metabolic pathways

The availability of bacterial prey had strong effects on the expression of genes involved in major carbon metabolic pathways. Higher glycolytic activity was implied by upregulated expression of genes coding for the unidirectional enzymes GCK and PFK in the presence of bacterial prey (i.e. upregulated in Dark + HKB or Light + HKB compared to Light + depleted HKB; Fig. 4). The expression of PEPCK, which connects the TCA cycle to glycolysis/gluconeogenesis, was also upregulated in the presence of bacterial prey. PEPCK normally converts oxaloacetate to phosphoenolpyruvate due to unfavorable kinetics of the reverse reaction [67, 68]. Upregulation of the gene for PEPCK in the presence of prey therefore likely indicates that succinic acid and oxaloacetate resulting from the degradation of bacterial amino acids were converted into phosphoenolpyruvate by PEPCK (Fig. 4) [69]. Conversely, carbon fixation from the Calvin cycle produces glyceraldehyde-3-P, which can be converted into phosphoenolpyruvate and fed into the TCA cycle as oxaloacetate through the activity of PEPC [70], an enzyme that catalyzes the formation of oxaloacetate from phosphoenolpyruvate (Fig. 4). Hence, upregulation of PEPC may be a reflection of higher carbon fixation activities. The gene coding for PEPC was indeed upregulated in the Light + depleted HKB treatment compared to Light + HKB (Additional file 6: Table S4).

Algae feeding on bacterial prey may generate excess metabolites in the TCA cycle that enter nitrogen metabolism pathways as oxoglutarate (Figs. 4 and 5). Genes associated with the TCA cycle leading to the formation of oxoglutarate in the present study were upregulated in the presence of bacterial prey (upregulated in Dark + HKB or Light + HKB compared to Light + depleted HKB), but genes for the oxoglutarate dehydrogenase complex (OGDC), which converts oxoglutarate to succinyl-CoA, were not differentially expressed between all 3 treatments (Fig. 4; Additional file 6: Table S4). This implied that oxoglutarate molecules exited the TCA cycle while the alga was performing phagotrophy. Oxoglutarate is a metabolite in nitrogen metabolism that functions as a transporter of nitrogen (Fig. 5) [71, 72].

Phagotrophy presumably also provided an ample supply of nitrogen to *Ochromonas* sp. strain BG-1 due to the nitrogen-rich nature of bacteria. Excess amino acids, including glutamate, are likely generated from the breakdown of bacterial proteins, and GLDH generates ammonium while converting glutamate into oxoglutarate (Fig. 5). The upregulated gene expression of GLDH in the presence of bacteria (upregulated in Light + HKB or Dark + HKB compared to Light + depleted HKB) is consistent with the documented release of ammonium in various species of *Ochromonas* (including strain BG-1) when the algae are feeding on bacteria [26, 52, 73]. Furthermore, the upregulation of AMTs in the Light + depleted HKB treatment compared to the Light + HKB or Dark + HKB treatments (Additional file 6: Table S4) is also consistent with previous records of ammonium uptake by *Ochromonas* species when light is available but prey are not [26, 52, 73].

### Genes of *Ochromonas* sp. strain BG-1 that are indicative of specific trophic modes

One of the goals of this study was to identify genes associated with specific trophic modes of *Ochromonas* sp. strain BG-1. For this reason, we investigated genes that were differentially expressed due to the availability of light (Light + HKB vs. Dark + HKB) or bacterial prey (Light + HKB vs. Light + depleted HKB). Genes with the highest fold changes in these two pairwise comparisons usually had no annotations, or were of unknown functions. However, genes for AMTs were amongst the most upregulated genes in the Light + depleted HKB treatment compared to Light + HKB (83 – 418 x fold change), and could potentially be markers for *Ochromonas* sp. strain BG-1 with high phototrophic activities (Additional file 6: Table S4).

In addition, there was a gene coding for an aureochrome-like protein that had the lowest relative expression in the presence of both light and prey. It was the second most upregulated gene in Dark + HKB compared to Light + HKB (25 x fold change) and was also strongly upregulated in Light + depleted HKB compared to Light + HKB (117 x fold change). Aureochromes are transcription factors with blue-light receptors that were first discovered in the xanthophyte, *Vaucheria frigida* [74]. While aureochromes have been detected in various *Ochromonas* species [16, 74], their physiological role(s) in these algae is still currently unclear [75]. Nevertheless, a low or lack of expression for this gene may be indicative of access to both light and prey (i.e. mixotrophic mode) for our studied alga.

### Ecological and evolutionary implications of mixotrophy in *Ochromonas* sp. strain BG-1

The ability of mixotrophic algae to perform both phototrophy and heterotrophy is presumed to incur both benefits and costs [4, 8]. *Ochromonas* sp. strain BG-1 appeared to gain little or no direct benefit from photosynthesis towards population growth in the presence of bacterial prey (Fig. 1). Therefore, the main benefit of retaining photosynthetic capabilities for the alga is most likely for survival when prey are not available. Phototrophy may also provide competitive advantage to the alga in the presence of heterotrophic competitors. In accordance with this hypothesis, Rothhaupt [76] demonstrated that light is required for an *Ochromonas* sp. to coexist with *Bodo* sp., a purely heterotrophic flagellate.

On the other hand, it is expected that there is a metabolic and energetic cost for mixotrophs to maintain both their cellular photosynthetic and heterotrophic machineries. While the estimated energetic cost for maintaining the photosynthetic machinery may be as high as 50% of the energy and nutrient requirements for cell synthesis in pure phototrophs [77], mixotrophs that are predominantly phagotrophic may have a lower cost (1 – 10%) because the photosynthetic apparatus does not occupy a large portion of the cell [78]. *Ochromonas* sp. strain BG-1 may further reduce the cost of phototrophy by limiting its light harvesting and photosynthetic activity when performing phagotrophy, as indicated by changes in gene expression and differences in cellular chlorophyll a contents demonstrated in this study. Low energetic cost for maintaining photosynthetic ability in strain BG-1 under phagotrophic nutrition is consistent with our observation that similar growth rates were observed for the alga growing with or without light when prey were available (Fig. 1).

Environmental conditions and resource availability may drive the evolution of mixotrophs towards stronger phagotrophic or phototrophic tendencies along the spectrum of mixotrophy [79]. As a result, it is conceivable that *Ochromonas* sp. strain BG-1 could lose its photosynthetic ability entirely if ample prey are constantly supplied. Bell [80], for example, was able to induce obligate osmotrophy in a once predominantly phototrophic green alga after several thousand generations of exclusive osmotrophic growth. Indeed, the loss of photosynthetic capabilities is common in various lineages [81], and phototrophy may be expendable to this *Ochromonas* species as long as prey are available because photosynthesis contributes negligibly to their population growth.

Nonetheless, even if a mixotrophic alga were to lose its photosynthetic capability entirely, it is unlikely that it would lose its chloroplast as the organelle is involved in other cellular metabolic processes in addition to photosynthesis. The C5 pathway for tetrapyrrole synthesis observed in *Ochromonas* sp. strain BG-1, for example, requires the chloroplast for the synthesis of not only chlorophyll but also heme groups [82]. Furthermore, isoprenoids and fatty acids are also produced in the chloroplast [83, 84]. Consequently, the chloroplast may be essential to general cell functioning even for organisms that had lost its photosynthetic capabilities. The complete loss of the chloroplast is rarely observed and difficult to prove [85].

## Conclusions

Our experiment detailed transcriptomic responses of *Ochromonas* sp. strain BG-1 to light despite a lack of differences in growth and bacterial grazing rates between algae growing in continuous light and dark conditions. The expression of a number of genes associated with photosynthesis, catabolic enzymes, as well as tetrapyrrole synthesis were upregulated when light was available. However, the availability of bacterial prey had a much higher impact on the alga and led to changes in the expression of most genes involved in major carbon and nitrogen metabolic pathways. Such changes implied higher glycolysis activities during phagotrophy and higher ammonium transport or uptake during phototrophy. Transcriptomic studies of mixotrophs thus improve our understanding of the metabolic processes occurring under different trophic modes, and reveal responses that does not necessarily lead to changes in population abundances.

## Additional files

Additional file 1: Table S1. The final concentration of components in the modified DY-V medium. (DOC 70 kb)
Additional file 2: Figure S1. Average natural logarithm of abundances (± SD) of *Ochromonas* sp. in different experimental treatments. Linear portion of the curve indicates the exponential growth period of *Ochromonas* sp. (JPG 825 kb)
Additional file 3: Table S2. A list of 14 stramenopile chloroplast genomes used to identify chloroplast genes in the *Ochromonas* sp. transcriptome. (DOC 81 kb)
Additional file 4: Figure S2. Non-metric multidimensional scaling (MDS) graph of the 9 RNA-Seq libraries of *Ochromonas* sp. The letter following the treatment name indicates the biological replicate ID (i.e. A-C). (JPG 97 kb)
Additional file 5: Table S3. Tables listing phototrophy-related genes (datasheet 1), phagotrophy-related genes (datasheet 2), and genes coding for glycoside hydrolases and lysozymes (datasheet 3). Blast2GO was used to obtained the GO or EC number(s) for each gene. (XLSX 49 kb)
Additional file 6: Table S4. Table listing genes associated with major carbon metabolic pathways (datasheet 1), major nitrogen metabolic pathways (datasheet 2), and tetrapyrrole synthesis (datasheet 3). Blast2GO was used to obtained the GO or EC number(s) for each gene. (XLSX 53 kb)
Additional file 7: Table S5. The abbreviations used in this study and the full names of enzymes involved in major carbon metabolic pathways, major nitrogen metabolic pathways, and tetrapyrrole synthesis. (DOC 113 kb)

## Abbreviations

DAPI: 4’ 6-diamidino-2-phenylindole dihydrochloride
FPKM: Fragment per kilobase of transcript per million reads
HKB: Heat-killed bacteria
TCA cycle: Tricarboxylic acid cycle
