# Supplementary figures and images for: Effect of light and prey availability on gene expression of the mixotrophic chrysophyte, Ochromonas sp
Source: BMC Genomics. 2017 Feb 14;18:163. doi: 10.1186/s12864-017-3549-1 (PMC5310065; doi:10.1186/s12864-017-3549-1)

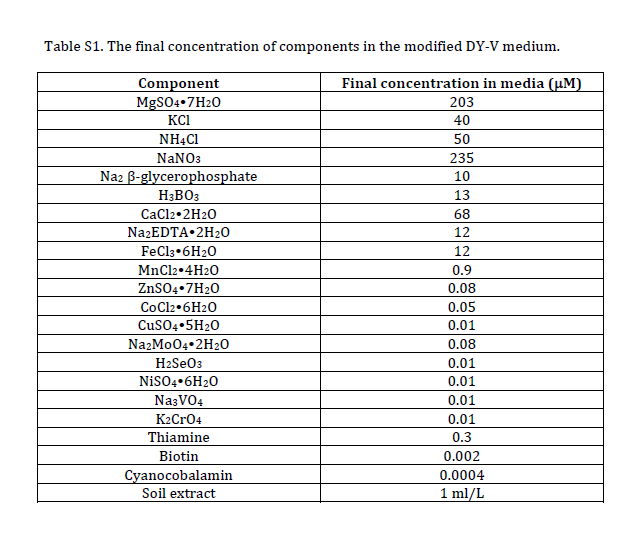

Supplement: Additional file 1: Table S1. — The final concentration of components in the modified DY-V medium. (DOC 70 kb) [file 12864_2017_3549_MOESM1_ESM.doc]

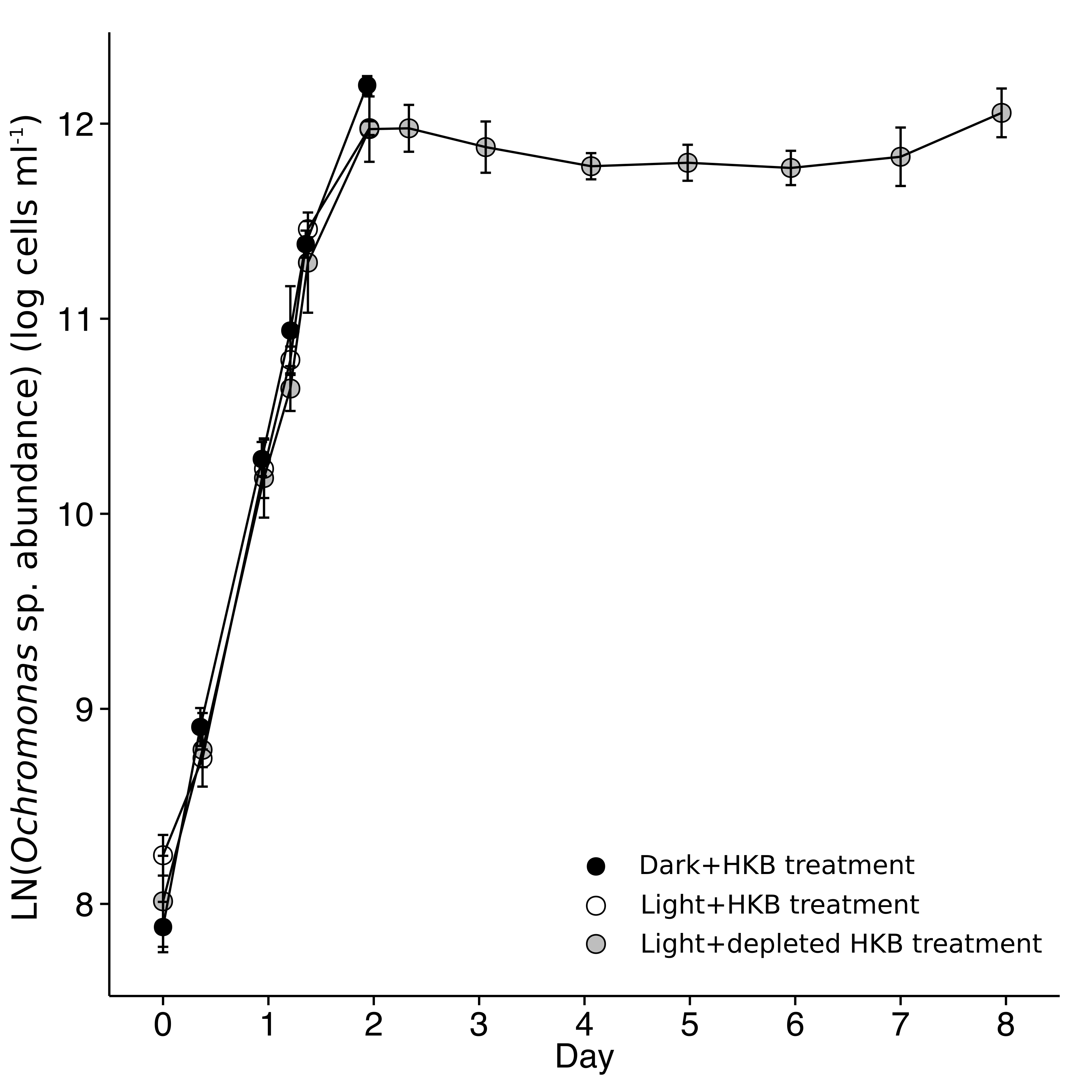

Supplement: Additional file 2: Figure S1. — Average natural logarithm of abundances (± SD) of Ochromonas sp. in different experimental treatments. Linear portion of the curve indicates the exponential growth period of Ochromonas sp. (JPG 825 kb) [file 12864_2017_3549_MOESM2_ESM.jpg]

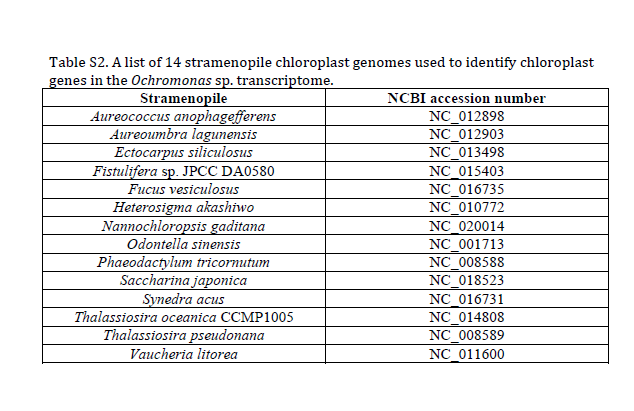

Supplement: Additional file 3: Table S2. — A list of 14 stramenopile chloroplast genomes used to identify chloroplast genes in the Ochromonas sp. transcriptome. (DOC 81 kb) [file 12864_2017_3549_MOESM3_ESM.doc]

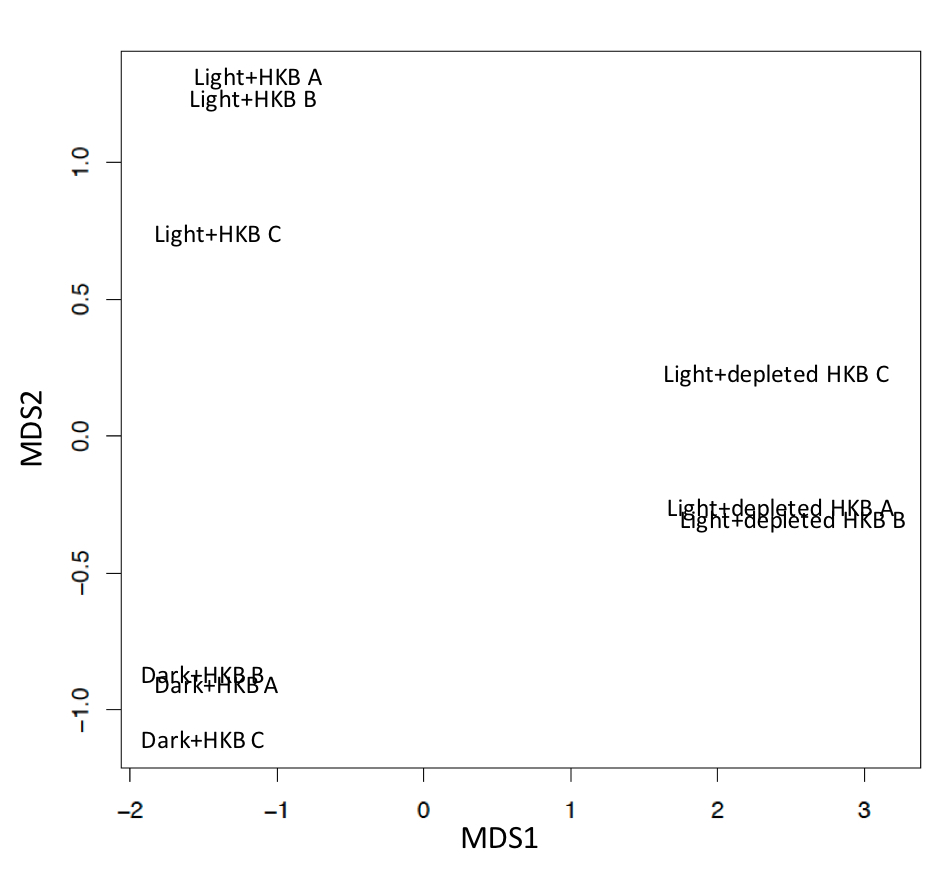

Supplement: Additional file 4: Figure S2. — Non-metric multidimensional scaling (MDS) graph of the 9 RNA-Seq libraries of Ochromonas sp. The letter following the treatment name indicates the biological replicate ID (i.e. A-C). (JPG 97 kb) [file 12864_2017_3549_MOESM4_ESM.jpg]
