# Supplementary material for: Effect of light and prey availability on gene expression of the mixotrophic chrysophyte, Ochromonas sp
Source: BMC Genomics. 2017 Feb 14;18:163. doi: 10.1186/s12864-017-3549-1 (PMC5310065; doi:10.1186/s12864-017-3549-1)
Supplement: Additional file 7: Table S5. — The abbreviations used in this study and the full names of enzymes involved in major carbon metabolic pathways, major nitrogen metabolic pathways, and tetrapyrrole synthesis. (DOC 113 kb) [file 12864_2017_3549_MOESM7_ESM.docx]

Table S5. The abbreviations used in this study and the full names of enzymes involved in major carbon metabolic pathways, major nitrogen metabolic pathways, and tetrapyrrole synthesis.

| **Pathway** | **Abbreviation** | **Full name** |
| --- | --- | --- |
| Major carbon metabolic pathways | ALD | Fructose-bisphosphate aldolase |
|  | CS | Citrate synthase |
|  | FBP | Fructose-1,6-bisphosphatase |
|  | GCK | Glucokinase |
|  | OGDC | Oxoglutarate dehydrogenase complex |
|  | PDC | Pyruvate dehydrogenase complex |
|  | PEPC | Phosphoenolpyruvate carboxylase |
|  | PEPCK | Phosphoenolpyruvate carboxykinase |
|  | PFK | Phosphofructokinase |
|  | PGI | Phosphoglucose isomerase |
|  | PK | Pyruvate kinase |
|  | PPDK | Pyruvate, phosphate dikinase |
| Major nitrogen metabolic pathways | AMT | Ammonium transporter |
|  | ARG | Arginase |
|  | ASL | Arginosuccinate lysase |
|  | ASS | Arginosuccinate synthase |
|  | GLDH | Glutamate dehydrogenase |
|  | GOGAT | Glutamine oxoglutarate aminotransferase |
|  | GS | Glutamine synthetase |
|  | OTC | Ornithine transcarbamylase |
|  | pgCPS | Pyrimidine synthesis glutamine carbamoyl phosphate synthase |
|  | unCPS | Urea ammonium carbamoyl phosphate synthase |
|  | URE | Urease |
| Tetrapyrrole synthesis | ALAD | 𝛅-aminolevulinic acid dehydratase |
|  | ChlG | Chlorophyll synthase |
|  | CPOX | Coproporphyrinogen oxidase |
|  | DVR | Divinyl chlorophyllide a 8-vinyl-reductase |
|  | FeCH | Ferrochelatase |
|  | FDBR | Ferredoxin-dependent bilin reductase |
|  | GluRS | Glutamyl-tRNA synthetase |
|  | GluTR | Glutamyl-tRNA reductase |
|  | GSA-AT | Glutamate 1-semialdehyde aminotransferase |
|  | HO | Heme oxygenase |
|  | MgCH | Magnesium chelatase |
|  | MgCy | Magnesium-protoporphyrin-IX-monomethyl ester cyclase |
|  | MgMT | Magnesium protoporphyrin IX methyltransferase |

Table S5 (continued).

| **Pathway** | **Abbreviation** | **Full name** |
| --- | --- | --- |
| Tetrapyrrole synthesis | PBGD | Porphobilinogen deaminase |
|  | POR | Light-dependent protochlorophyllide reductase |
|  | PPOX | Protoporphyrinogen oxidase |
|  | PS | Phytochromobilin synthase |
|  | Siroheme FeCH | Siroheme ferrochelatase |
|  | UROD | Uroporphyrinogen decarboxylase |
|  | UROM | Uroporphyrinogen methylase |
|  | UROS | Uroporphyrinogen synthase |
